# Delta (B1.617.2) variant of SARS-CoV-2 induces severe neurotropic patterns in K18-hACE2 mice

**DOI:** 10.1038/s41598-023-29909-x

**Published:** 2023-02-27

**Authors:** Ju-Hee Yang, Myeon-Sik Yang, Dae-Min Kim, Bumseok Kim, Dongseob Tark, Sang-Min Kang, Gun-Hee Lee

**Affiliations:** 1 Laboratory for Infectious Disease Prevention, Korea Zoonosis Research Institute, Jeonbuk National University, Iksan, 54531 Republic of Korea; 2 Laboratory of Veterinary Pathology, The College of Veterinary Medicine, Jeonbuk National University, Iksan, 54596 Republic of Korea

**Keywords:** SARS-CoV-2, Zoology

## Abstract

A highly contagious virus, severe acute respiratory syndrome coronavirus 2, caused the coronavirus disease 19 (COVID-19) pandemic (SARS-CoV-2). SARS-CoV-2 genetic variants have been reported to circulate throughout the COVID-19 pandemic. COVID-19 symptoms include respiratory symptoms, fever, muscle pain, and breathing difficulty. In addition, up to 30% of COVID-19 patients experience neurological complications such as headaches, nausea, stroke, and anosmia. However, the neurotropism of SARS-CoV-2 infection remains largely unknown. This study investigated the neurotropic patterns between the B1.617.2 (Delta) and Hu-1 variants (Wuhan, early strain) in K18-hACE2 mice. Despite both the variants inducing similar pathogenic patterns in various organs, B1.617.2-infected K18-hACE2 mice demonstrated a higher range of disease phenotypes such as weight loss, lethality, and conjunctivitis when compared to those in Hu-1-infected mice. In addition, histopathological analysis revealed that B1.617.2 infects the brain of K18-hACE2 mice more rapidly and effectively than Hu-1. Finally, we discovered that, in B1.617.2-infected mice, the early activation of various signature genes involved innate cytokines and that the necrosis-related response was most pronounced than that in Hu-1-infected mice. The present findings indicate the neuroinvasive properties of SARS-CoV-2 variants in K18-hACE2 mice and link them to fatal neuro-dissemination during the disease onset.

## Introduction

A novel, highly contagious virus termed severe acute respiratory syndrome coronavirus 2 (SARS-CoV-2) emerged in December 2019 and swiftly expanded globally^1^. Coronavirus disease 19 (COVID-19) primarily affects the respiratory system and various organs such as the brain^2^. In addition, a high proportion of COVID-19 patients exhibit neurological manifestations such as hypogeusia, dizziness, headaches, myalgia, impaired consciousness, hyposmia, and ataxia^3,4^. In the early pandemic, 36.4% of patients in Wuhan, China exhibited neurological symptoms, with 8.9% having peripheral nervous system symptoms, including anosmia (5.1%)^5^. Multiple lines of evidence demonstrated that SARS-CoV-2-induced brain damage-induced neurological symptoms are frequent and disabling events^6–8^. These findings suggested that SARS-CoV-2 infection can infiltrate the central nervous system (CNS) and the respiratory system. However, the mechanism by which SARS-CoV-2 invades the CNS is unclear. ACE2 and TMPRSS2, which are recognized as SARS-CoV-2 receptors for infection and dissemination in the body, are expressed at limited levels in the brain relative to that in the lungs^9^. However, early studies reported that neuropilin-1 (NRP-1), basigin, cathepsin L, and furin can enhance SARS-CoV-2 invasiveness and are more highly expressed in the brain than ACE2 or TMPRSS2^10–12^. Several studies have revealed that the SARS-CoV-2 neuroinvasive mechanism involves hematological dissemination across a disrupted blood–brain barrier (BBB) and direct CNS infection^13–15^. In addition, SARS-CoV-2 infection-induced inflammatory responses cause BBB dysfunction, facilitating viral entry into the CNS^16^. Although the mechanism of SARS-CoV-2 neuroinvasion is unclear, considering the similar genetic characteristics of SARS-CoV, a similar neuroinvasion mechanism for SARS-CoV-2 may exist^17^.

SARS-CoV-2, like other RNA viruses, has evolved rapidly and accumulated mutations in its viral genome through frequent recombination and evolutionary adaptation, giving rise to multiple variants of concern^18^. The B1.617.2 (delta) variant emerged in India in October 2020, and it exhibited nearly twofold higher infectivity than the early strain (Wuhan, Hu-1) during its predominant circulation period of June–December 2021^19^. Despite the unknown etiology, in-hospital death rate was higher for patients infected with B1.617.2 (vaccinated or unvaccinated cases) than for those infected with the other strains^20^. Furthermore, immune escape mutations of 13 amino acids in the spike (S) protein, including the receptor-binding domain, enhance viral infectivity, viral particle production^21^, and stability^22^. We also expected to observe pathogenic differences between patients infected with B1.617.2 and the early strain in the brain and respiratory system. In this study, we assessed the Hu-1 and B1.617.2 respiratory symptoms and the neurotropic patterns in K18-hACE2 mice, which are susceptible to SARS-CoV-2 infection, and often demonstrate COVID-19–like disease symptoms^23^. Our studies clarified the variant’s lethality and histopathology, as well as changes in various cellular response genes after infection of the brains or lungs of the K18-hACE2 mice. These observations can help clarify the pathogenic characteristics and the neurotropic patterns of B1.617.2 and provide insights into the potential treatment responses for COVID-19 patients.

## Results

### The B1.617.2 variant is more virulent than the early strain in the brains of K18-hACE2 mice

**Figure 1:**
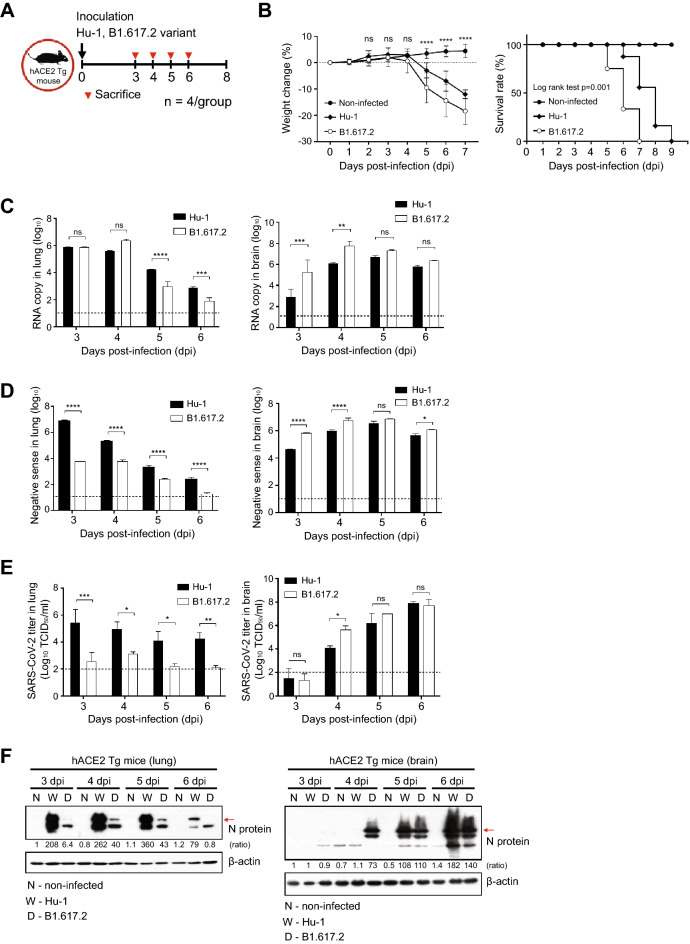
The B1.617.2 variant is more virulent than the Hu-1 strain in K18-hACE2 mice. The schematic illustration of the animal experiment. K18-hACE2 mice were intranasally inoculated with the Hu-1 strain or B1.617.2 variant (**A**). Weight loss and mortality were monitored in K18-hACE2 mice after inoculation with the indicated strains (**B**). Representative data were analyzed from three independent experiments and presented as the mean ± SEM value (n = 12). The viral burden (**C**) and negative-sense strand (**D**) in the lungs and brains were quantified by qRT-PCR at the indicated times after infection. The titration of SARS-CoV-2 in the lungs and brains was performed by TCID_50_ for the infectious virus (**E**). Western blotting detected the N protein of SARS-CoV-2 in the lung and brain homogenates at the indicated times after infection (**F**). Two-way ANOVA was performed, followed by Dunnett’s multiple-comparison tests. Statistical significance is indicated by asterisks (n = 5, *p < 0.05, **p < 0.01, ***p < 001, ****p < 0.0001, *ns* not significant). C, Control; H, Hu-1; B, B1.617.2. The viral RNA copies and infectious SARS-CoV-2 limit of detection were log_10_/copies and 2 log_10_ TCID_50_/mL, respectively (dot line). The image J software (version 1.53 k, http://imagej.nih.gov/ij/) normalized the relative viral N protein expression with β-actin.

Intranasally, we administered 2.5 × 10^4^ of 50% tissue culture-infectious dose (TCID_50_)/mL SARS-CoV-2, Hu-1 (early strain), or B1.617.2 (delta variant) into 7-week-old heterozygous K18-hACE2 mice (Fig. 1A). The resultant clinical symptoms were monitored for 8 days post-infection (dpi). K18-hACE2 mice infected with B1.617.2 exhibited more severe weight loss (> 20%) and an earlier onset of lethality in mice (death at day 5) than those infected with the Hu-1 strain (death at day 6). Both viruses infected mice showed distinguished 100% lethality by day 7 in the B1.617.2 variant and, by day 9, in the Hu-1 strain (Fig. 1B). Furthermore, K18-hACE2 mice infected with B1.617.2 frequently exhibited inflammation throughout the eye, but in the Hu-1 infection group, inflammation was only observed in the corners of the eyes (Sup. Fig. S1A). The autopsy revealed damage in several organs including severe hemorrhage in the brain, lungs, and spleen (Sup. Fig. S1B–D), albeit no discernable injury in the kidneys (Sup. Fig. S1E) or other organs. These clinical lesions of the brain and lungs differed between mice inoculated with B1.617.2 and Hu-1; specifically, B1.617.2-infected mice showed severe brain and mild lung damage, whereas the Hu-1–infected mice showed severe lung and moderate brain damage. At 3–6 dpi, we assessed the viral burden in the lung and brain homogenates. B1.617.2 revealed similar trends in reducing viral RNA copies and subgenomic RNA when compared to Hu-1. The lower levels of viral RNA (Fig. 1C), subgenomic RNA (Fig. 1D), and infectious SARS-CoV-2 (Fig. 1E) were detected in B1.617.2-infected lungs over time, whereas a significant difference showed at only 4 dpi in the B1.617.2-infected brains. At 6 dpi, the viral RNA levels in other tissues, including the heart, kidneys, and spleen, were similar between B1.617.2- and Hu-1–infected lungs. By contrast, no viral RNA was detected in the liver or trachea (Sup. Fig. S2). The expression of hACE2, a SARS-CoV-2 receptor, was constant, supporting SARS-CoV-2 infection in the brain and other tissues (Sup. Fig. S3). During SARS-CoV-2 infection, hACE2 expression was lower in the lungs than in the brains (Sup. Fig. S4). Similarly, the expression of the nucleocapsid (N) protein declined in both Hu-1–and B1.617.2-infected lungs with time. At 4 dpi, viral N protein was detected in the B1.617.2-infected brain (Fig. 1F). These data suggested that the B1.617.2 variant infects the brain earlier than the Hu-1 strain and disseminates more rapidly, which may be associated with the early onset of clinical symptoms.

### Differences of neuropathological complications in the brains of K18-hACE2 mice after SARS-CoV-2 infection

**Figure 2:**
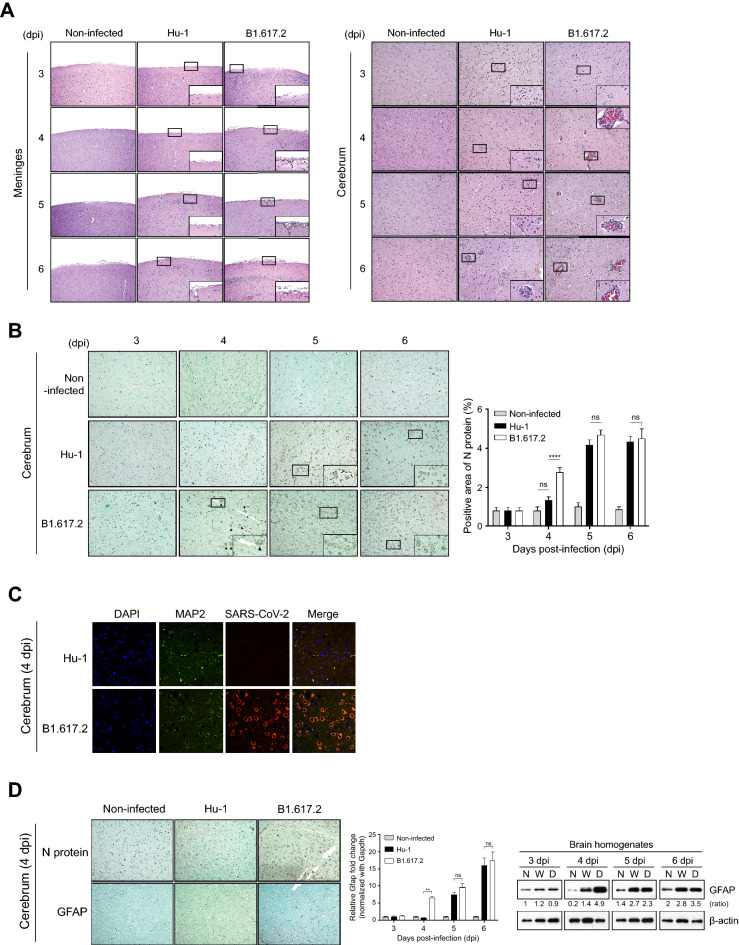
Histopathological analysis of SARS-CoV-2–infected K18-hACE2 mice. The abnormalities of the brain sections stained with hematoxylin and eosin were observed after the SARS-CoV-2 infection (**A**). The N protein level was detected in the brain sections and cerebrum at the indicated times after infection (left panel), and the N protein-positive area was quantified (right panel) (**B**). Representative immunofluorescence images of 4 dpi brain sections infected with SARS-CoV-2 display the localization of N protein (SARS-CoV-2, red) and MAP2 (neuronal cells, green) (**C**). GFAP, a marker of astrocytes, was stained in the brain sections at 4 dpi (left panel), and its levels were quantified by qRT-PCR (middle panel, n = 5) and Western blotting (right panel) at the indicated time after infection (**D**). Two-way ANOVA with Dunnett’s multiple-comparison test was performed to determine the significance (**p < 0.01, ns; not significant). Images are representative of four images per group. Magnification, × 200. C, Control; H, Hu-1; B, B1.617.2. The relative GFAP expression was normalized with β-actin by using the Image J software (version 1.53 k, http://imagej.nih.gov/ij/).

We assessed histopathological changes in hematoxylin and eosin-stained lung (Sup. Fig. S5) and brain sections (Fig. 2) from Hu-1–or B1.617.2-infected K18-hACE2 mice. The histopathological score was calculated using a microscopic grading system as the average of the representative severity of the lungs (Sup. Table S1). At 3 dpi, we observed similar lung pathology in both variants, including moderate pulmonary edema and infiltrating inflammatory cells in the perivascular and peribronchial regions with progressive inflammation. At 4 dpi, these patterns accelerated lung consolidation, infiltrating inflammatory cells, and a partial loss of bronchiole epithelial cilia in the Hu-1–infected lung sections. At 6 dpi, Hu-1 and B1.617.2-infected K18-hACE2 mice displayed consolidation in 35%–50% of the lungs and blood leakage from vessels into the adjacent alveolar space and alveolar wall thickening (Sup. Fig. S5). Furthermore, we observed the brain pathology in both SARS-CoV-2-infected brain tissue, including the meninges and cerebrum. At 4 dpi, the B1.617.2-infected brain sections displayed an increase in microglia and radial glia cell counts (Sup. Fig. S6) in the adjacent meningeal vessels and the perivascular region of the cerebrum, as well as a partial detachment of the meninges. Nonetheless, these changes were not present in the Hu-1–infected brain sections. B1.617.2 infection continuously increased the infiltrating glial cell numbers, culminating in meningeal disruption at 6 dpi (Fig. 2A and Sup. Fig. S6). Conversely, the predominant histopathological changes of Hu-1–infected brain sections were generally weak inflammatory responses that developed as late-onset symptoms when compared to the findings in B1.617.2-infected brain sections. To determine whether brain damage was correlated with the severity of SARS-CoV-2 infection, we stained the brain sections with immunohistochemistry for the SARS-CoV-2 N protein. At 4 dpi, N protein was distributed predominantly in perivascular neuronal cells in the B1.617.2-infected cerebrum sections, but not detected in the Hu-1–infected cerebrum sections (Fig. 2B). At 5 and 6 dpi, SARS-CoV-2 infection of neuronal cells widely spread throughout the cerebrum (Fig. 2B). The cells infected with SARS-CoV-2 were then stained with a neuron-specific marker known as microtubule-associated protein 2 (MAP2) to demonstrate that they were neuronal cells. At 4 dpi, the brain section infected with B1.617.2 demonstrated the co-localization of N protein (SARS-CoV-2) with MAP2 (neuronal cell) compared to the Hu-1-infected brain section (Fig. 2C). Furthermore, the same pattern was detected in the cerebrum sections stained with GFAP, an astrocyte marker, and glial fibrillary acidic protein (GFAP). At 4 dpi, the viral RNA and protein levels were significantly increased in B1.617.2-infected brains (Fig. 2D). These data suggested that brain damage caused by SARS-CoV-2 infection recruited multinucleated cells including microglia, radial glia, and astrocytes. In addition, the neuronal cells were more infected and sensitive to the B1.617.2 variant than the Hu-1 strain.

### The early cellular responses to SARS-CoV-2 infection

**Figure 3:**
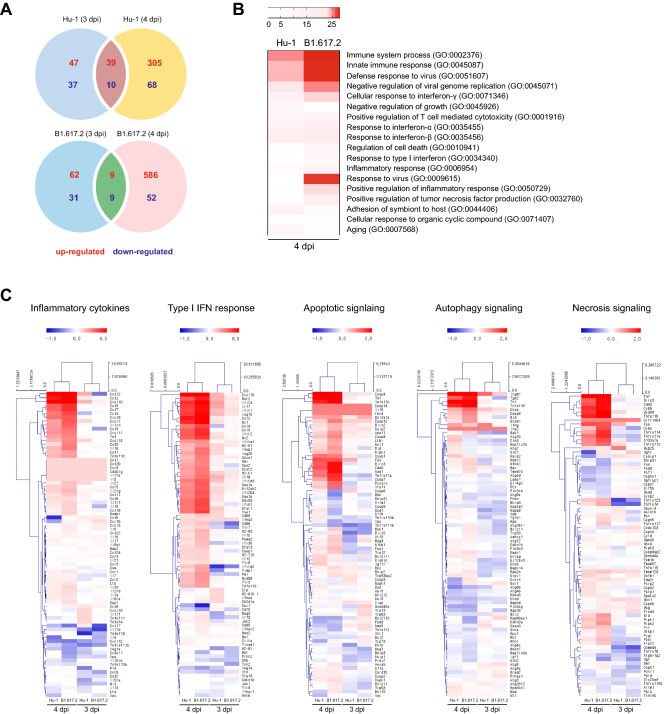
SARS-CoV-2 infection-induced multi-transcriptional signatures associated with various cellular responses. The NGS data of the brain homogenates of 3 and 4 dpi. Venn diagram indicated the overlapping genes with differential expression. The total number of significantly upregulated and downregulated genes when compared to the corresponding findings in the non-infected mice (**A**). Biological responses enriched in differentially expressed genes were analyzed by Gene Ontology at 4 dpi when compared to the corresponding findings in the non-infected mice. The false discovery process (q value) determined the rank, and the indicated responses are listed after eliminating the redundant genes (**B**). Heat maps indicating upregulated genes at 3 and 4 days after Hu-1 or B1.617.2 infection. The cellular signal pathways with enriched inflammatory cytokines, type-I IFN responses, apoptosis, autophagy, and necrosis signaling were identified by Gene Ontology (**C**). The differentially expressed genes presented in each cellular pathway are the combinations of differential expressed genes. Based on the RNA-seq database, the gene ontology and Heat maps were generated using GraphPad Prism 7.0 (https://www.graphpad.com/).

To assess how the kinetics of infection and the subsequent processes modulate the early cellular response to SARS-CoV-2 in the brain, we performed the Next generation sequencing (NGS) of SARS-CoV-2-infected brain homogenates at 0 (control), 3, and 4 dpi. The Venn diagram in Fig. 3A depicts the upregulated and downregulated genes in SARS-CoV-2–infected brain homogenates at 3 and 4 dpi as compared to that in the control animals. The enrichment of gene signatures in Hu-1–infected brain homogenates demonstrated an increase in the number of upregulated (from 47 to 305 genes) and downregulated genes (from 37 to 68 genes), with only 49 genes overlapping at 3 and 4 dpi. Conversely, upregulated (from 62 to 586 genes), downregulated (from 31 to 52 genes), and overlapped (18 genes) gene signatures were increased in B1.617.2-infected homogenates. Gene Ontology analysis of the top-upregulated genes identified various cellular responses, such as the immune system processes, innate immune responses, inflammation responses, viral defense responses, and interferon (IFN) responses (Fig. 3B). These findings highlighted the regulation of gene sets involved in type-I IFN signaling, inflammatory cytokine signaling, and glial cell migration. An hiPSC-derived neuronal organoid study reported dysregulated inflammatory and innate immune responses coupled with cell-death regulation^24^. At 4 dpi, we revealed that inflammatory cytokine-associated genes (*Ccl5*, *Ccl7*, *Cxcl1*, *Il1b*, and *Csf3*), Type-I IFN-associated genes (*Irf7*, *Stat1*, and *Oas2*), and certain IFN-stimulated genes (*Ifit1*, *Oas2*, *Mx2*, and *Irf7*) were upregulated in the B1.617.2-infected tissues compared to those in the Hu-1–infected tissues (Fig. 3C). In addition, the cell-death process was activated earlier in the B1.617.2-infected brains than in the Hu-1–infected brains. SARS-CoV-2 infection upregulated the expression of genes involved in apoptotic (*Casp8*, *Casp7*, and *Nod1*) and necrotic (*Tnf*, *Ngfr*, *Ripk1*, *Ripk3*, and *Pygl*) processes. An in vitro study reported that SARS-CoV-2 limits autophagy signaling and inhibits autophagic flux^25^. Similarly, the expression of autophagy-associated genes (*Becn1* and *Atg7*) was neither altered nor reduced, indicating that an autophagy-independent cell-death program was activated in the SARS-CoV-2–infected brain (Fig. 3C). These distinct transcriptional changes were determined as temporary occurrences in the early stage of SARS-CoV-2-infected brain, indicating immune-associated or cellular-regulated characteristics.

### The necrotic pathway activated during the early brain response to SARS-CoV-2 infection

**Figure 4:**
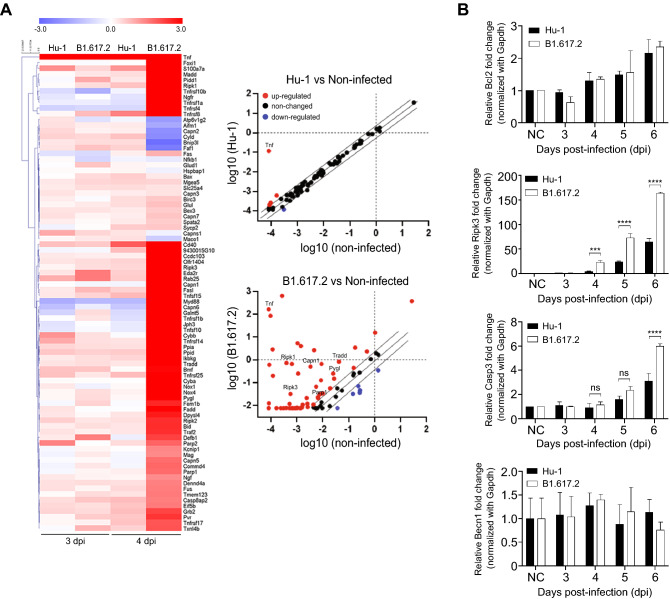
Necroptosis-associated genes were induced in early-stage brain homogenates during B1.617.2 infection. The brain homogenates obtained at 3 and 4 dpi were analyzed simultaneously to profile 84 necrosis genes by using the RT^2^ profiler PCR assay. The heat maps present the upregulated genes in the brain homogenates (left panel), and a scatter plot reveals the upregulated (red), downregulated (blue), and unchanged (black) genes (**A**). The plot presents the log tenfold changes in gene expression in the two groups. The quantification of the cell death-related genes *Bcl2* (pro-apoptotic), *Ripk3* (necroptosis), *Casp3* (apoptotic), and *Becn1* (autophagic) in the brain homogenates at the indicated time points after infection (**B**). Based on the GeneGlobe database (https://dataanalysis2.qiagen.com/pcr), the heat map for the RT^2^-profiler PCR array was generated using GraphPad Prism 7.0 (https://www.graphpad.com/).

We investigated the commitment of the necrotic process in the early brain response after SARS-CoV-2 infection. The mouse necrosis RT^2^-profiler PCR array assessed the expression of necrosis-related genes in the brain homogenates infected with SARS-CoV-2 at 3 and 4 dpi. At 4 dpi, unsupervised clustering analysis illustrated that most necrosis-related genes were expressed at higher levels in B1.617.2-infected brains than in Hu-1–infected brains (Fig. 4A, left). Necrosis-related genes were upregulated (Hu-1: 4 and 4; B1.617.2: 4 and 62) and downregulated (Hu-1: 1 and 2; B1.617.2: 1 and 6) after 3 and 4 days of infection, respectively. The indicated genes included necrotic markers such as receptor-interacting kinase 1 (*Ripk1*), *Ripk3*, glycogen phosphorylase L (*Pygl*), Poly (ADP-ribose) polymerase 1 (*Parp1*), and a calpain-1 catalytic subunit (*Capn1*) (Fig. 4A, right). In addition, we identified a significant difference in the *Ripk3* expression across SARS-CoV-2 infection groups. However, *Bcl2* (pro-apoptotic), *Casp3* (apoptotic), and *Becn1* (autophagy) expression did not differ between the groups (Fig. 4B). These findings suggest that SARS-CoV-2–infected neuronal cells can promote a Ripk3-dependent cell-death program in the early brain response.

## Discussion

The B1.617.2 variant was first identified in Maharashtra, India, and it carries three key mutations (i.e., L452R, T478K, and P681R) in the receptor-binding motif of S protein. These alterations rapidly became dominant globally because of the increased virus infectivity and the reported evasion of neutralizing antibodies, which is associated with its transmissibility^26–29^. Moreover, the epidemiological characteristics of the B1.617.2 variant included higher risks of hospitalization, intensive care unit admission, and mortality when compared to that observed for N501Y-positive variants and the early strain^30–32^. K18-hACE2 mice consistently expressed hACE2, thereby promoting systemic virus dissemination in most of the tissues and enhancing the infectivity of SARS-CoV-2. Several infected mice had a significant error value of the viral burden and titration following the change of hACE2 expression because of SARS-CoV-2–induced hACE2 downregulation or cell death in the infected tissues^33,34^. Since K18-hACE2 mice exhibited enhanced neurotropism, which was not detected in patients due to the overexpression of hACE2 in all epithelial tissues, they have limitations in that they do not accurately represent the disease phenotype observed in humans. Nevertheless, K18-hACE2 mice were considered an appropriate model for studying the lethal cases of COVID-19. In addition, SARS-CoV-2–infected K18-hACE2 mice exhibited neurological signs such as circling, rolling, and flaccid paralysis of the hind legs, which eventually led to death^23^. Moreover, it was previously reported that the potential replication of SASR-CoV-2 in neuronal cells could have lethal consequences in the CNS of K18-hACE2 mice^35^. In this study, we investigated the pathological changes and temporal changes of host factors involved in cellular and inflammatory responses during SARS-CoV-2 (Hu-1 or B1.617.2) infection in K18-hACE2 mice. As the B1.617.2 variant was associated with high infectivity and mortality, we expected this variant to cause more extensive changes in the clinical indices of the virus-infected brain relative to that by Hu-1. As expected, B1.617.2-infected mice displayed high lethality, neurological signs, severe hemorrhage, and weight loss when compared to the corresponding findings in Hu-1-infected mice. However, the B1.617.2 variant tends to feature the opposite patterns in terms of the viral burden, infectious virus titer, and viral replication, as well as the N protein levels in the infected lungs and brains. SARS-CoV-2 infection in the lungs leads to hACE2 downregulation, hACE2 shedding, or death in the hACE2-expressing cells^36,37^. We also demonstrated that the hACE2 levels were decreased in the lungs following SARS-CoV-2 infection, which suggests the high virulence of B1.617.2 in the pneumocytes and the possibility that the variant promotes inflammatory processes associated with hACE2 imbalance on the cell surface. Histopathological analysis revealed that SARS-CoV-2 infection caused the disruption of meninges and the infiltration of inflammatory cells, but no ischemia in the brain and lungs^35,38,39^.

The distinct mechanism of SARS-CoV-2 infection in the brain remains unclarified. The possible mechanisms include SARS-CoV-2 infection in the olfactory nerve, vascular endothelial cell infection, and invasion through inflammation-induced disruption of the BBB^40^. A few studies have reported that SARS-CoV-2 targets neurons and neuronal progenitors for subsequent replications^14,41^. Our results indicated that the patterns of neuron and reactive astrocyte infection, known as gliosis, caused by Hu-1 or B1.617.2 differed among the K18-hACE2 mice. We attributed this difference to the possible susceptibility of the brain by neuropilin-1 (NRP1), which enhances the SARS-CoV-2 entry^10^, resulting in sustained brain damage and enhanced severity.

Cytokine analysis of the brains of K18-hACE2 mice infected with Hu-1 or B1.617.2 revealed markedly different cell-death profiles, including apoptosis and necrosis, whereas the inflammatory and innate immune responses were similar between the groups. SARS-CoV-2–mediated regulation of the cell-death pathways has been reported in several cell types and neuronal cells^40,42^. The cell death processes can cooperate and they are often accompanied by the activation of multicellular factors, which implies the activation of defenses against intracellular infection. In addition, these processes can promote innate and adaptive immune responses and inflammatory responses, which act synergistically to regulate cell fate^43^. Ripk1 and Ripk3 are the key factors that regulate necrotic cell death. Under virus infection, which promotes Ripk1-dependent necrosis by stimulating tumor necrosis factor (TNF), Ripk3 subsequently induced the phosphorylation of Ripk1, which resulted in the formation of a pro-necrotic necrosome complex. In addition, Ripk3 phosphorylates mixed lineage kinase domain-like pseudokinase (MLKL), which is distributed on the plasma membrane, demonstrating necroptosis activation, and it also interacts with metabolic enzymes, such as Pygl, which contributes to ROS production and necroptosis^44^. Our results revealed that B1.617.2 induced the expression of necrosis- and apoptosis-related genes including *Ripk1*, *Ripk3*, *Tradd*, *Pygl*, *Fadd*, *Il-1β*, and *Casp3* when compared to the corresponding findings in Hu-1-infected mice. Necroptosis and apoptosis are programmed forms of cell death that are activated by SARS-CoV-2 infection^45,46^. Our results demonstrated that the expression of the necroptosis marker RIPK3 was increased by B1.617.2 at the early time when compared with that after Hu-1 infection; moreover, the mortality was earlier in mice infected with B1.617.2. In addition, the apoptosis marker caspase-3 was increased after 6 days of infection with B1.617.2 when compared to that in the Hu-1-infected mice. These results can serve as evidence for analyzing the causes of why B1.617.2 is classified as variants of concern and fatal symptoms in people. In our subsequent studies, we aim to clarify the mechanism by which SARS-CoV-2 induces necroptosis and cell-death responses in the brain. Furthermore, whether necrotic factors are conserved in the brains of patients infected with SARS-CoV-2 variants is an interesting question for future studies. Finally, our study findings can facilitate the clarification of the pathogenic characteristics of the B1.617.2 variant and identify the potential factors that control brain damage and improve the survival outcomes of patients with COVID-19.

## Methods

### Virus and cells

The Hu-1 (BetaCoV/Korea/KCDC03/2020, NCCP43326) and B1.617.2 strains (hCoV-19/Korea/KDCA119861/2021, NCCP43390) were obtained from the Korea Disease Control and Prevention Agency. African green monkey kidney epithelial cells (Vero E6, ATCC CRL-1586) were cultured in Dulbecco’s modified Eagle’s medium (DMEM, Thermo Fisher Scientific, Waltham, MA, USA) supplemented with 10% fetal bovine serum (FBS, Thermo Fisher Scientific), 100 U/mL penicillin, and 100 µg/mL streptomycin (Thermo Fisher Scientific). The propagation and titration of SARS-CoV-2 in the Vero E6 cells were calculated using TCID_50_, as described previously^1^. Briefly, Vero E6 cells were infected with the B1.617.2 (2.9 × 10^6^ TCID_50_/mL) or Wuhan strain (1 × 10^6^ TCID_50_/mL), and the cytopathic effect (CPE) was monitored at 3–5 dpi. The experiments associated with SARS-CoV-2 infection were performed at a biosafety level 3 laboratory with the use of personal protection equipment in accordance with the biosafety manual instructions issued by the Korea Zoonosis Research Institute of Jeonbuk National University.

### Mouse experiment

Heterozygous K18-hACE2 mice [strain: JAX 034,860 B6.Cg-Tg(K18-hACE2)2Prlmn/J] were purchased from the Jackson Laboratory (Bar Harbor, ME, USA). Seven-week-old male K18-hACE2 mice were administrated 2.5 × 10^4^ TCID_50_/mL SARS-CoV-2 via the intranasal route. The K18-hACE2 mice were monitored for changes in weight loss, lethality, and clinical symptoms every day after inoculation. At 6 dpi, SARS-CoV-2–infected K18-hACE2 mice were sacrificed by isoflurane and autopsied to assess the clinical lesions in several organs such as the brain, heart, lungs, spleen, and kidneys. The animal experiments were approved by the Institutional Animal Committee of the Jeonbuk National University (JBNU 2020-11-001) and performed in accordance with the guidelines of the Institutional Biosafety Committee. The study was conducted in compliance with the ARRIVE guidelines.

### Measurement of the viral burden and viral protein levels in K18-hACE2 mouse tissues

Different tissues of SARS-CoV-2–infected K18-hACE2 mice were obtained by autopsy and then homogenized in a 2-mL homogenous tube (Bertin Technologies SAS, France) with RIPA lysis buffer or TRIzol (Invitrogen, Carlsbad, CA, USA) after treatment with RNA protect-tissue reagent (Qiagen, Venlo, Netherlands). Total RNA was purified as per a commercial manual, and, subsequently, cDNA was synthesized using an all-in-one master mix (Cellsafe, Yongin, South Korea) for 5 min at 25 °C, for 60 min at 42 °C, and 5 s at 85 °C. The target genes were quantified by qRT-PCR with the IQ SYBR Green (Bio-Rad, Seoul, South Korea) using target-specific primer sets. The supernatants were collected from homogenized tissues in the RIPA lysis buffer. The quantification of total protein was performed by using a BCA protein assay kit (Thermo Fisher Scientific) and subjected to SDS-PAGE, followed by Western blotting with specific antibodies. The protein was detected by developing (Poohung, Kyunggi, South Korea) into X-ray films (AGFA, Mortsel, Belgium) using an ECL kit (ELPIS, Daejeon, South Korea). The western blot images comply with the digital and integrity policy (the full, unprocessed images are included in the supplementary information file S1). The target-specific primer sets and primary antibodies are described in Supplementary Table S2.

### Histopathological and immunohistochemical analyses

SARS-CoV-2–infected brain and lung tissues were fixed in 10% formalin solution (Sigma–Aldrich, St. Louis, MO, USA) and then embedded in paraffin wax (Leica Biosystems, Wetzlar, Germany). Formalin-fixed, paraffin-embedded tissue blocks were sectioned at a thickness of 4 μm with an HM 340 electronic rotary microtome (Thermo Fisher Scientific). The tissue sections were then stained with hematoxylin and eosin as per the standard laboratory protocol^47^, and the pulmonary abnormalities were scored based on the representative microscopic lesions. The severity of each criterion was scored 0–3 as described in Supplementary Table S1. As the lesions were not uniformly distributed and different patterns were detected in the tissues, caution was practiced when scoring. The scores for each criterion were summed, with the higher scores indicating more severe damage. For immunohistochemistry, the sections were mounted onto silane-coated slides and treated with citrate buffer (pH 6.0) at 95 °C for 30 min and room temperature for 20 min. The sections were incubated overnight with SARS-CoV-2 nucleocapsid protein (Sino Biological, China), MAP2 (Invitrogen, Carlsbad, CA, USA), and GFAP antibody (Cell Signaling Technology, CA, USA) at 4 °C. Each slide was washed thrice for 15 min each with the wash buffer (0.145 M NaCl, 0.0027 M KCl, 0.0081 M Na_2_HPO_4_, 0.0015 M KH_2_PO_4_, pH 7.4 in PBS). The sections were incubated with horseradish peroxidase-conjugated anti-rabbit IgG (Vector Laboratories, CA, USA). The antibodies were visualized with 3,3′-diaminobenzidine (Vector Laboratories) in accordance with the manufacturer’s instructions. The histopathological examinations were performed in a double-blinded manner by trained pathologists. To quantify the immunohistochemistry outcomes, the images were randomly captured from each stained tissue and analyzed by the TS Auto 5.1 (Olympus, Tokyo, Japan). The percent immunohistochemistry-positive area was analyzed in a defined magnification field and area (magnification, × 200; field, 0.144 mm^2^).

### TCID50 assay

SARS-CoV-2–infected K18-hACE2 mouse lungs or brains were homogenized with PBS. The clarified supernatants were collected via centrifugation and then serially diluted with the DMEM without serum. Vero E6 cells (3 × 10^4^ cells/well) were inoculated with four replicates from 1 × 10^−8^ to 1 × 10^−1^ diluents. The diluents were then removed, and the medium was sequentially replaced with DMEM supplemented with 2% FBS. At 3–5 dpi, CPE was monitored, and TCID_50_ of SARS-CoV-2 was calculated by the Reed and Muench method^48^.

### RNA-seq analysis

Total RNA was isolated and quantified with the Bioanalyzer 2100 (Agilent Technologies, CA, USA). Redundant ribosomal RNA (rRNA) was eliminated from total RNA using the RiboCop rRNA Depletion Kit (Lexogen, Vienna, Australia). The RNA-seq libraries were prepared using the Next Ultra II Directional RNA kit (NEB, MA, USA) according to the manufacturer’s protocol. The libraries were pooled and analyzed as paired-end sequenced on the NovaSeq 6000 (Illumina, CA, USA) targeting 40 million read pairs and extended. The RNA-seq reads were then aligned to the mouse reference genome (mm10) with the TopHat. All gene counts were preprocessed with the EdgeR to adjust the samples for differences in the library size using the trimmed mean of M values. The results of the differential signature genes were analyzed with the ExDEGA (eBiogen, Seoul, South Korea). Gene Ontology analysis and classification were performed using the data from the Database for Annotation, Visualization, and Integrated Discovery.

### RT2-profiler PCR array

Total RNA extracted from SARS-CoV-2–infected K18-hACE2 mouse brains at 3 and 4 dpi was synthesized using the RT^2^ First Strand Kit (Qiagen, Hilden, Germany). The synthesized cDNA was mixed with RT^2^ SYBR Green Mastermix, and the RT^2^ profiler™ PCR array mouse necrosis pathway (Qiagen, PAMM-141ZA/330231). The qPCR array was performed by holding for 10 min at 95 °C, followed by 40 cycles of 15 s at 95 °C and 60 s at 60 °C. The result was then analyzed with GeneGlobe (https://dataanalysis2.qiagen.com/pcr), and the Ct values were normalized with the supplied internal housekeeping gene.

## Supplementary Information

Supplementary Information 1.

Supplementary Information 2.

Supplementary Information 3.

## Data Availability

All data generated during this study are included in the supplementary material files. RNA-seq data that supported the present findings have been deposited in the NCBI GEO (accession number: GSE214516; https://www.ncbi.nlm.nih.gov/geo/query/acc.cgi?acc=GSE214516).

## References

[1] Roberts, D. L., Rossman, J. S. & Jarić, I. Dating first cases of COVID-19. *PLoS Pathog.* **17**, e1009620. 10.1371/journal.ppat.1009620 (2021). 10.1371/journal.ppat.1009620 PMC8224943 34166465

[2] Puelles VG (2020). Multiorgan and renal tropism of SARS-CoV-2. N. Engl. J. Med..

[3] Nuzzo D (2021). Post-acute COVID-19 neurological syndrome: A new medical challenge. J. Clin. Med..

[4] Acharya A (2020). SARS-CoV-2 infection leads to neurological dysfunction. J. Neuroimmune Pharmacol..

[5] Mao L (2020). Neurologic manifestations of hospitalized patients with coronavirus disease 2019 in Wuhan China. JAMA. Neurol..

[6] Marshall M (2020). How COVID-19 can damage the brain. Nature.

[7] Pleasure SJ, Green AJ, Josephson SA (2020). The spectrum of neurological disease in the severe acute respiratory syndrome coronavirus 2 pandemic infection: Neurologists move to the frontlines. JAMA Neurol..

[8] Chen, T., *et al*. Clinical characteristics of 113 deceased patients with coronavirus disease 2019: Retrospective study. *BMJ* **368**, m1091. 10.1136/bmj.m1091 (2020). 10.1136/bmj.m1091 PMC7190011 32217556

[9] Hammin, I., *et al*. Tissue distribution of ACE2 protein, the functional receptor for SARS coronavirus. A first step in understanding SARS pathogenesis. *J. Pathol.* **203**, 631–637. 10.1002/path.1570 (2004). 10.1002/path.1570 PMC7167720 15141377

[10] Cantuti-Castelvetri L (2020). Neuropilin-1 facilitates SARS-CoV-2 cell entry and infectivity. Science.

[11] Coutard, B., *et al*. The spike glycoprotein of the new coronavirus 2019-nCoV contains a furin-like cleavage site absent in CoV of the same clade. *Antiviral Res.* **176**, 104742. 10.1016/j.antiviral.2020.104742 (2020). 10.1016/j.antiviral.2020.104742 PMC7114094 32057769

[12] Davies J (2020). Neuropilin-1 as a new potential SARS-CoV-2 infection mediator implicated in the neurologic features and central nervous system involvement of COVID-19. Mol. Med. Rep..

[13] Krasemann S (2020). The blood-brain barrier is dysregulated in COVID-19 and serve as a CNS entry route for SARS-CoV-2. Stem Cell Rep..

[14] Ramani, A., *et al*. SARS-CoV-2 targets neurons of 3D human brain organoids. *EMBO J.* **39**, e106230. 10.15252/embj.2020106230 (2020). 10.15252/embj.2020106230 PMC7560208 32876341

[15] Pennisi M (2020). SARS-CoV-2 and the nervous system: from clinical features to molecular mechanisms. Int. J. Mol. Sci..

[16] Guadarrama-Ortiz P (2020). Neurological aspects of SARS-CoV-2 infection: Mechanisms and manifestations. Front. Neurol..

[17] Li Y-C, Bai W-Z, Hashikawa T (2020). The neuroinvasive potential of SARS-CoV-2 may play a role in the respiratory failure of COVID-19 patients. J. Med. Virol..

[18] Burki T (2022). The origin of SARS-CoV-2 variants of concern. Lancet Infect. Dis..

[19] Liu, Y., & Rocklöv, J. The reproductive number of the Delta variant of SARS-CoV-2 is far higher compared to the ancestral SARS-CoV-2 virus. *J. Travel. Med.* **28**, taab124. 10.1093/jtm/taab124 (2021). 10.1093/jtm/taab124 PMC8436367 34369565

[20] Lauring, A. S., *et al*. Clinical severity of, and effectiveness of mRNA vaccines against, covid-19 from omicron, delta, and alpha SARS-CoV-2 variants in the United States: prospective observational study. *BMJ*. **376**, e069761. 10.1136/bmj-2021-069761 (2022). 10.1136/bmj-2021-069761 PMC8905308 35264324

[21] Callaway E (2021). The mutation that helps Delta spread like wildfire. Nature.

[22] Liu Z (2021). Identification of SARS-CoV-2 spike mutations that attenuate monoclonal and serum antibody neutralization. Cell Host Microbe..

[23] Yinda, C. K., *et al.* K18-hACE2 mice develop respiratory disease resembling severe COVID-19. *PLoS Pathog.* **17**, e1009195 (2021). 10.1371/journal.ppat.1009195 10.1371/journal.ppat.1009195 PMC7875348 33465158

[24] Jacob F (2020). Human pluripotent stem cell-derived neural cells and brain organoids reveal SARS-CoV-2 neurotropism predominates in choroid plexus epithelium. Cell Stem Cell.

[25] Gassen NC (2021). SARS-CoV-2-mediated dysregulation of metabolism and autophagy uncovers host-targeting antivirals. Nat. Commun..

[26] Seeley JJ (2018). Induction of innate immune memory via microRNA targeting of chromatin remodelling factors. Nature.

[27] Harvey WT (2021). SARS-CoV-2 variants, spike mutations and immune escape. Nat. Rev. Microbial..

[28] Deng X (2021). Transmission, infectivity, and neutralization of a spike L452R SARS-CoV-2 variant. Cell.

[29] Giacomo SD (2021). Preliminary report on severe acute respiratory syndrome coronavirus 2 (SARS-CoV-2) spike mutation T478K. J. Med. Virol..

[30] Fisman DN, Tuite AR (2021). Evaluation of the relative virulence of novel SARS-CoV-2 variants: A retrospective cohort study in Ontario Canada. CMAJ.

[31] Callaway E (2021). Delta coronavirus variant: Scientists brace for impact. Nature.

[32] Sheikh A (2021). SARS-CoV-2 Delta VOC in Scotland: Demographics, risk of hospital admission, and vaccine effectiveness. Lancet.

[33] Winkler ES (2020). SARS-CoV-2 infection of human ACE2-transgenic mice causes severe lung inflammation and impaired function. Nat. Immunol..

[34] Kuba K (2005). A crucial role of angiotensin converting enzyme 2 (ACE2) in SARS coronavirus-induced lung injury. Nat. Med..

[35] Song, E., *et al.* Neuroinvasion of SARS-CoV-2 in human and mouse brain. *J. Exp. Med.* **218**, e20202135 (2021). 10.1084/jem.20202135 10.1084/jem.20202135 PMC7808299 33433624

[36] Wang, J., Zhao, H. & An, Y. ACE2 shedding and the role in COVID-19. *Front. Cell Infect. Microbiol.* **11**, 789180 (2021). 10.3389/fcimb.2021.789180 10.3389/fcimb.2021.789180 PMC8795668 35096642

[37] Vaduganathan M (2020). Renin-Angiotensin-Aldosterone system inhibitors in patients with Covid-19. N. Engl. J. Med..

[38] Vidal E (2022). Chronological brain lesions after SARS-CoV-2 infection in hACE2-transgenic mice. Vet. Pathol..

[39] Chavda V (2022). Ischemic stroke and SARS-CoV-2 infection: The bidirectional pathology and risk morbidities. Neurol. Int..

[40] Wan D (2021). Neurological complications and infection mechanism of SARS-CoV-2. Signal Transduct. Target Ther..

[41] Zhang B-Z (2020). SARS-CoV-2 infects human neural progenitor cells and brain organoids. Cell Res..

[42] Bader SM (2022). Programmed cell death: The pathways to severe COVID-19?. Biochem. J..

[43] Bedoui S, Herold MJ, Strasser A (2020). Emerging connectivity of programmed cell death pathways and its physiological implications. Nat. Rev. Mol. Cell Biol..

[44] Humphries F (2015). RIP kinases: Key decision makers in cell death and innate immunity. Cell Death Differ..

[45] Fritsch M (2019). Caspase-8 is the molecular switch for apoptosis, necroptosis and pyroptosis. Nature.

[46] Li S (2020). SARS-CoV-2 triggers inflammatory responses and cell death through caspase-8 activation. Signal Transduct. Target Ther..

[47] Yang M-S (2021). Ultra- and micro-structural changes of respiratory tracts in SARS-CoV-2 infected Syrian hamsters. Vet. Res..

[48] Reed LJ, Muench H (1938). A simple method of estimating fifty percent endpoints. Am. J. Epidemiol..

