# Author Correction: Delta (B1.617.2) variant of SARS-CoV-2 induces severe neurotropic patterns in K18-hACE2 mice

**DOI:** 10.1038/s41598-023-30977-2

**Published:** 2023-03-06

**Authors:** Ju-Hee Yang, Myeon-Sik Yang, Dae-Min Kim, Bumseok Kim, Dongseob Tark, Sang-Min Kang, Gun-Hee Lee

**Affiliations:** 1 Laboratory for Infectious Disease Prevention, Korea Zoonosis Research Institute, Jeonbuk National University, Iksan, 54531 Republic of Korea; 2 Laboratory of Veterinary Pathology, The College of Veterinary Medicine, Jeonbuk National University, Iksan, 54596 Republic of Korea

Correction to: *Scientific Reports* 10.1038/s41598-023-29909-x, published online 27 February 2023

The Acknowledgments section in the original version of this Article was incomplete.

“This research was supported by the Basic Science Research Program through the National Research Foundation of Korea (NRF) funded by the Ministry of Education (2021R1C1C2005307 and 2017R1A6A1A03015876).”

now reads:

“This research was supported by the Basic Science Research Program through the National Research Foundation of Korea (NRF) funded by the Ministry of Education (2021R1C1C2005307 and 2017R1A6A1A03015876). It was also supported by the grant of the Korea Health Industry Development Institute (KHIDI, HI22C1637).”

The original Article has been corrected.

