# Supplementary material for: Delta (B1.617.2) variant of SARS-CoV-2 induces severe neurotropic patterns in K18-hACE2 mice
Source: Sci Rep. 2023 Feb 27;13:3303. doi: 10.1038/s41598-023-29909-x (PMC9970970; doi:10.1038/s41598-023-29909-x)

Supplementary. Figure 1

Clinical symptoms of SARS-CoV-2 infected K18-hACE2 mice

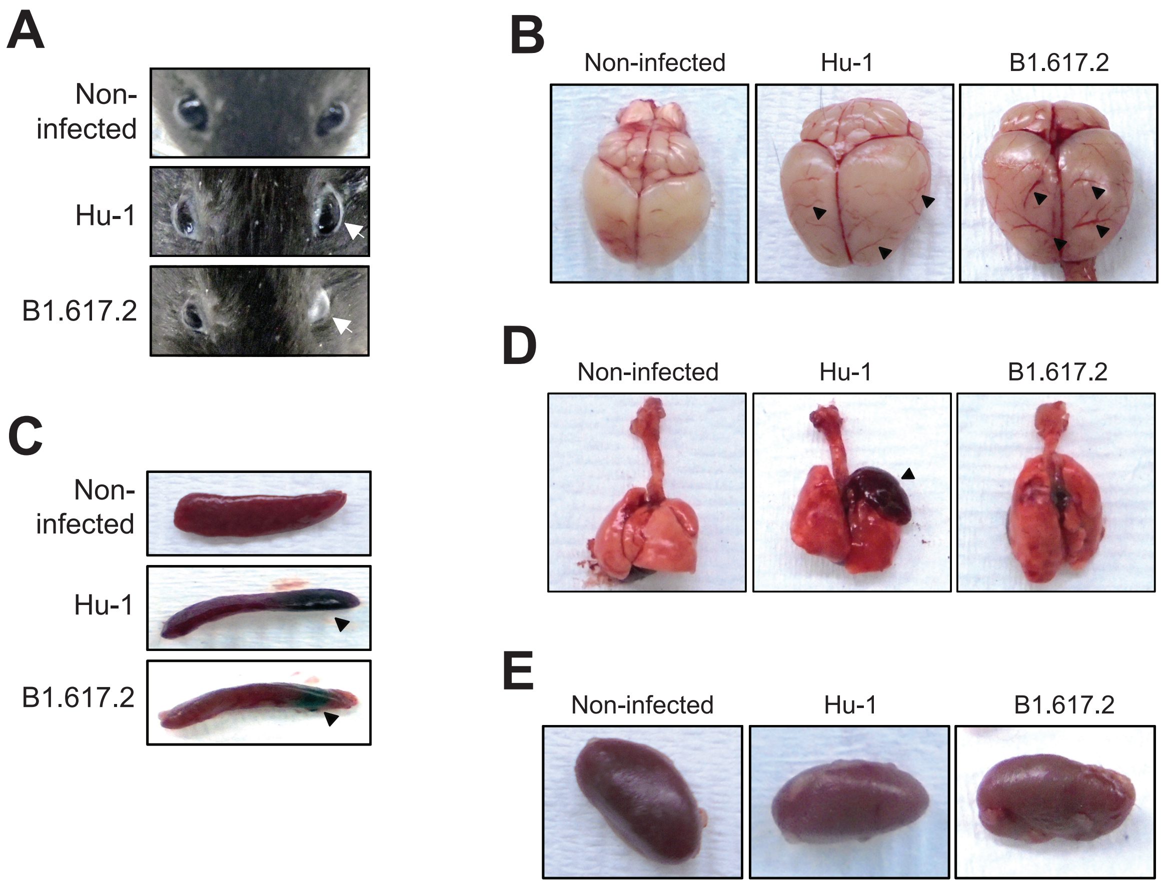

Supplementary. Figure 2

Viral RNA levels in various tissue of SARS-CoV-2 infected K18-hACE2 mice

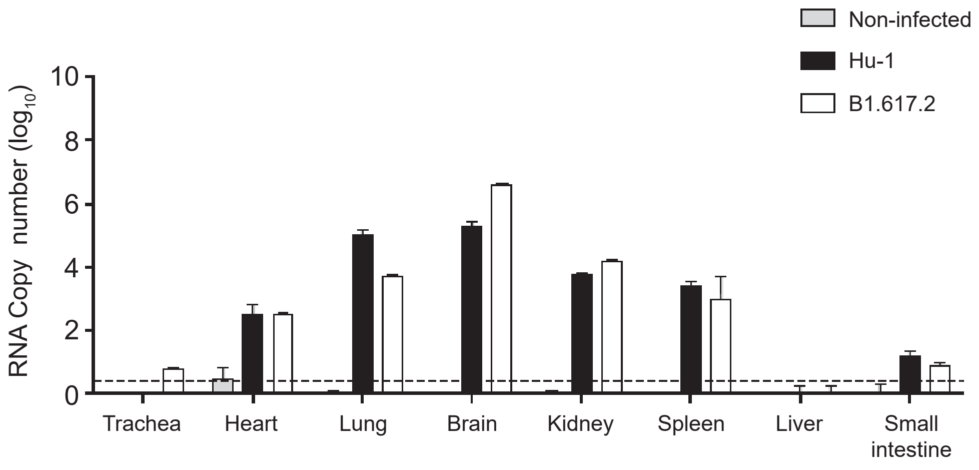

Supplementary. Figure 3

hACE2 expression levels in various tissues of K18-hACE2 mice

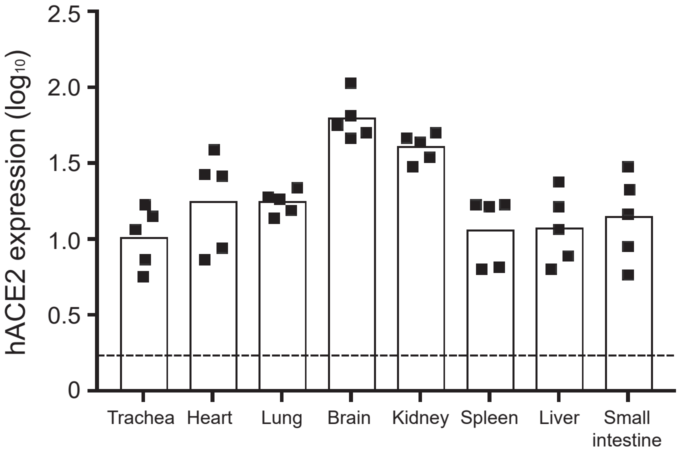

Supplementary. Figure 4

hACE2 levels in infected lungs and brains of K18-hACE2 Tg mice

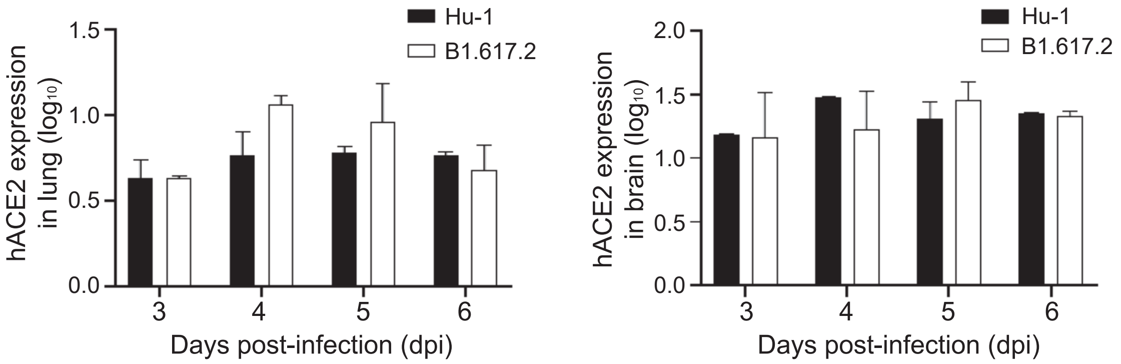

Supplementary. Figure 5

Histopathological analysis of lung section in SARS-CoV-2 infected hACE2 mice

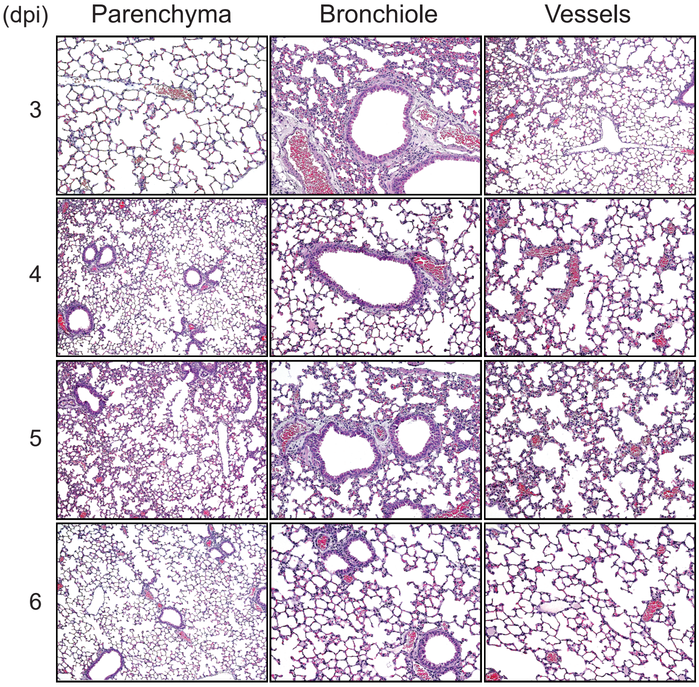

Non-infected

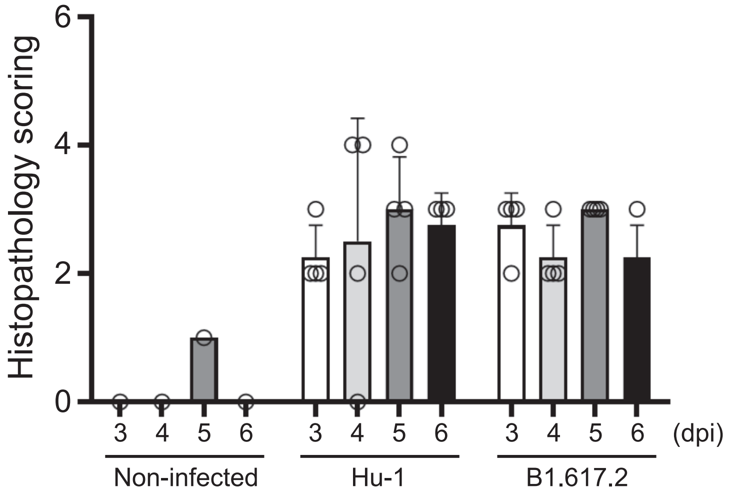

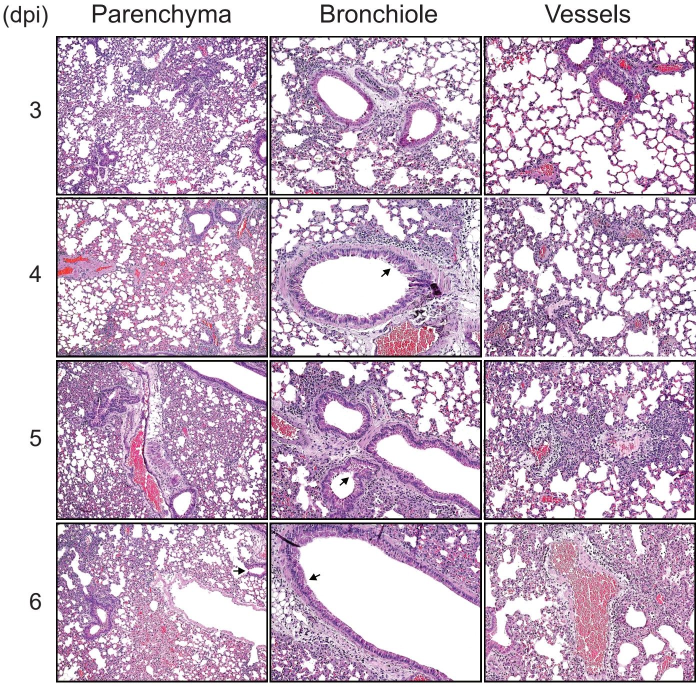

Hu-1

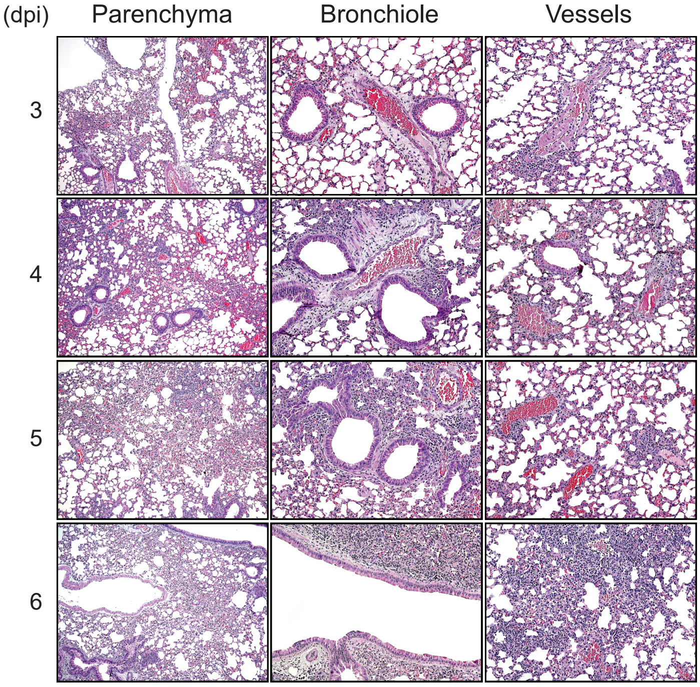

B1.617.2

Supplementary. Figure 6

Glial cell increased by SARS-CoV-2 infection

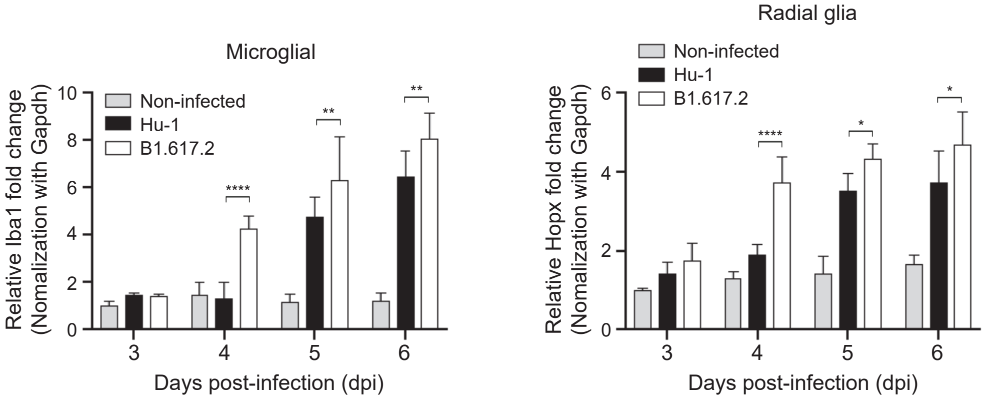

Supplement: Supplementary file 1 — Supplementary Information 1. [file 41598_2023_29909_MOESM1_ESM.pdf]
