# Supplementary material for: Delta (B1.617.2) variant of SARS-CoV-2 induces severe neurotropic patterns in K18-hACE2 mice
Source: Sci Rep. 2023 Feb 27;13:3303. doi: 10.1038/s41598-023-29909-x (PMC9970970; doi:10.1038/s41598-023-29909-x)
Supplement: Supplementary file 2 — Supplementary Information 2. [file 41598_2023_29909_MOESM2_ESM.docx]

**Supplementary Figure Legends**

Supplementary. Figure 1. Clinical symptoms of SARS-CoV-2–infected K18-hACE2 mice. K18-hACE2 mice were intranasally inoculated with 2.5 × 10^4^ TCID_50_/ml Hu-1 or B1.617.2. Infected mice were monitored for tissue damage. At 5 dpi, infected mice displayed eye inflammation (A). The infected brains (B), spleens (C), and lungs (D) with hemorrhage were observed after autopsy, but phenotypes of kidneys were normal (E).

Supplementary. Figure 2. Viral RNA levels in various tissues from SARS-CoV-2–infected K18-hACE2 mice. The levels of viral RNA in homogenates of the indicated tissues were quantified by qRT-PCR at 6 dpi (n = 3). The limited of detection indicated a dotted line.

Supplementary. Figure 3. hACE2 expression in various tissues from K18-hACE2 mice. hACE2 levels were quantified in homogenates from non-infected tissues by qRT-PCR at 6 dpi (n = 5). The detection of hACE2-negative mice indicated a dotted line.

Supplementary. Figure 4. hACE2 levels in the infected lungs and brains of K18-hACE2 mice. hACE2 levels were quantified in lung (left) and brain (right) homogenates by qRT-PCR at the indicated time after infection (n = 3).

Supplementary. Figure 5. Histopathological analysis of the lung sections of SARS-CoV-2–infected hACE2 mice. The lung sections were stained with hematoxylin and eosin after SARS-CoV-2 infection or mock infection. The pulmonary abnormalities were scored using the specified scoring criteria. Images are representative of five images per group, and three different regions are presented. Magnification: parenchyma, ×100; bronchioles and vessels, ×200.

Supplementary. Figure 6. The recruitment of glial cells was increased by SARS-CoV-2 infection. The microglial (left) and radial glia (right) mRNA levels were quantified in brain homogenates using specific primer sets as Iba1 and Hopx at the indicated time after infection (n = 3). To determine the significance performed two-way ANOVA with Dunnett’s multiple comparison tests (*p < 0.05, **p < 0.01, ****p < 0.0001).
