# Supplementary material for: Delta (B1.617.2) variant of SARS-CoV-2 induces severe neurotropic patterns in K18-hACE2 mice
Source: Sci Rep. 2023 Feb 27;13:3303. doi: 10.1038/s41598-023-29909-x (PMC9970970; doi:10.1038/s41598-023-29909-x)
Supplement: Supplementary file 3 — Supplementary Information 3. [file 41598_2023_29909_MOESM3_ESM.docx]

| Supplementary. Table 1. The parameters for histopathologic examination scoring | | | |
| --- | --- | --- | --- |
| Organ | Type of lesions | | Representative lesions |
| Lung | Inflammation | Peribronchiolar | - Inflammatory cell infiltration |
|  |  |  | - Parabronchiolar inflammation |
|  |  | Perivascular | - Inflammatory cell infiltration |
|  |  |  | - Hyperemia |
|  |  | Bronchiole lumen | - Protein-rich exudate |
|  |  |  | - Cellular exudate |
|  |  |  | - Floating detached epithelial cell |
|  |  |  | - Infiltrate into alveolar space |
|  | Bronchiole epithelium damage | | - Morphological change of epithelial  Cell |
|  |  |  | - Loss of epithelial cilia |
|  |  |  | - Detached epithelium from basement membrane |
|  | Interstitial pneumonia | | - Thickened alveolar wall |
|  |  |  | - Inflammatory cell infiltration into alveolar wall |
|  |  |  | - Proportion of lesions |
|  | Hemorrhage | | - Blood leakage from vessels into |
|  |  |  | Perivascular region |
|  |  |  | Alveolar space |
| * Each parameter has a range of 0–3 regarding the severity or proportion | | | |
| ** 0, normal or <10%; 1, mild or 10%–40%; 2, moderate or 40%–70%; 3, severe or >70% | | | |
| *** The average score represents the severity in each organ | | | |

| Supplementary. Table 2. Primer sets and antibodies used in this study | | |  |
| --- | --- | --- | --- |
| Gene | Forward sequence | Reverse sequence | Reference |
| Human ACE2 | TCCATTGGTCTTCTGTCACCCG | AGACCATCCACCTCCACTTCTC | *Oncotarget* **11**, 4201 (2020) |
| N gene (SARS-CoV-2) | TAATCAGACAAGGAACTGATTA | CGAAGGTGTGACTTCCATG | *Nature* **583**, 459-468 (2020) |
| N gene (antisense) | GTTCCCGAAGGTGTGACTTC | GGGGACCAGGAACTAATCAGAC | This study |
| RdRp gene (SARS-CoV-2) | AGAATAGAGCTCGCACCGTAG | CTCCTCTAGTGGCGGCTATT | *Mar. Drugs*. **20**, 296 (2022) |
| GFAP | ACCTGCAGATTCGAGAAACC | CTCCTTAATGACCTCTCCATCC | *Cancer* **9**, 4496 (2018) |
| Ripk3 | AAGTGCAGATTGGGAACTACAACTC | AGAATGTTGTGAGCTTCAGGAAGTG | *Parasites & vectors* **10**, 1-19 (2017) |
| Gapdh | CATCACTGCCACCCAGAAGACTG | ATGCCAGTGAGCTTCCCGTTCAG | *Biochem. Biophys. Res. Commun.* **496**, 1197-1203 (2018) |
| Bcl2 | GTGGATGACTGAGTACCTGAACC | AGCCAGGAGAAATCAAACAGAG | *Parasites & Vectors* **10**, 1-19 (2017) |
| Casp3 | AGCAGCTTTGTGTGTGTGATTCTAA | AGTTTCGGCTTTCCAGTCAGAC |  |
| Becn1 | TGATCCAGGAGCTGGAAGAT | CAAGCGACCCAGTCTGAAAT |  |
| Iba1 | GAAGCGAATGCTGGAGAAAC | AAGATGGCAGATCTCTTGCC | *Front Cell Neurosci* **11**,129 (2017) |
| Hopx | TTCAACAAGGTCAACAAGCACCCG | CCAGGCGCTGCTTAAACCATTTCT | *Nature Communications* **6**, 6727 (2015) |

| Antibody | Manufacturer | Source | Catalog No. |
| --- | --- | --- | --- |
| Nucleocapsid (SARS-CoV-2) | Sino Biological | Rabbit | 40143-T62 |
| GFAP | Cell Signaling | Rabbit | 80788 |
| Beta-actin | Cell Signaling | Rabbit | 4967 |
| Anti-mouse IgG, HRP-linked | Cell Signaling | Horse | 7076 |
| Anti-rabbit IgG, HRP-linked | Cell Signaling | Goat | 7074 |
| MAP2 | Invitrogen | Mouse | MA5-12826 |
| Alexa 488 goat anti-mouse IgG | Invitrogen | Goat | A-11001 |
| Alexa 555 goat anti-rabbit | Invitrogen | Goat | A-21428 |
| DAPI | Vector |  | H-1200 |
